# Health-Related Consequences of Work-Family Conflict From a European Perspective: Results of a Scoping Review

**DOI:** 10.3389/fpubh.2019.00189

**Published:** 2019-07-09

**Authors:** Lea-Sophie Borgmann, Petra Rattay, Thomas Lampert

**Affiliations:** Department of Epidemiology and Health Monitoring, Robert Koch Institute, Berlin, Germany

**Keywords:** work-to-family conflict, family-to-work conflict, self-rated health, mental health, physical health, scoping review, Europe

## Abstract

**Background:** Rising percentages of working mothers and increasing numbers of dual-earner couples are putting work-family conflicts on the agenda. Studies based on data from the US have already proven a link between work-family conflict and health in working parents with heterogeneous results for certain health outcomes and subgroups. Also, to date no comprehensive overview of the existing evidence regarding the impact of work-family conflict on health among European working parents exist.

**Methods:** A scoping review was conducted to identify and analyze knowledge gaps regarding health-related consequences of work-family conflicts. To search for relevant publications on work-family conflicts and health, a systematic prospective literature search was carried out in two international databases (PubMed and Scopus) based on four landmark publications. The search was complemented by a systematic retrospective search in Scopus and hand searches. Inclusion criteria were a focus on work-family conflict, an analysis of health-related outcomes, and the presentation of empirical results. The publications were summarized in narrative style.

**Results:** A total of *n* = 25 publications on work-family conflict and health in Europe were identified. The data suggests that a variety of instruments is used to measure work-family conflict. Also, work-family conflict and health are linked in Europe, although longitudinal data do not always show robust causal interrelations. Most studies focus on self-rated, mental, and physical health. Results for gender-specific health outcomes remain controversial.

**Conclusion:** The review provides an overview of existing evidence for health-related consequences of work-family conflicts in Europe. The results of the review strengthen the evidence for a link between work-family conflict and health. However, heterogeneous results regarding the direction of work-family conflict and high-risk groups are a matter for discussion. This study investigates whether differences in the results can be accounted for by diverse measurement methods and study populations. Furthermore, different family policies in the European region as well intersectional approaches should be taken into account in further research.

## Introduction

### Background and State of Research

The reconciliation of work and family life is a current topic of international political and public debates (1, 2). An increasing labor force participation of women over the past two decades in Europe led to a rising share of women and men having to reconcile employment and family work (3). Scientific interest in conflicts emerging from the reconciliation of work and family roles began in the mid-1990s, driven primarily by scientists in the USA (4). The most common term used is “work-family conflict,” which is defined as an inter-role conflict in which the demands of the work and family roles are not mutually compatible (5). The resulting conflicts can have impact in two directions: work-to-family conflicts occur when the demands of employment disturb family life; family-to-work conflicts arise when family demands interfere with the job role (5). In addition to the directions of conflict, a distinction is also made between types of conflict: time, strain, behavior, and energy (5, 6). Work-family conflicts are defined as a specific form of work-life conflicts (7).

In addition to more women in the labor markets, developments such as digitalization and globalization lead to changing workplaces and new demands from employment (3). At the same time, employees face the challenge of organizing care for their children and, as societies are aging, also having to provide care for relatives in need (3, 8). Particularly for parents, rising workplace and familial flexibilities—via more flexible working-hours and an increasing availability of institutional childcare—offer opportunities to improve the compatibility of work and family roles. But new conflicts are emerging as boundaries between employment and family work become blurred and the demands of both areas overlap. The resulting work-family conflicts can, in turn, create a range of strains for employees and working parents in particular.

In addition to work-family conflicts, the reconciliation of multiple roles can also results in so called work-family enrichment or work-life balance (9–13). However, besides the importance of this perspective the conceptualizations and measuring instruments in this field have not yet reached the same level as for work-family conflicts (12).

Work-family conflict is measured using a variety of validated scales that measure different directions and types of conflicts (12). The majority of these scales were validated in the US-American region, making the comparability and interpretation of results, for example on the basis of European data, considerably more difficult (15). To date, no overview of scales used in Europe exists.

With regard to consequences, work-family conflicts can deteriorate the ability to work and increase the likelihood of having an impaired family life. However, the most frequently mentioned consequence of work-family conflict is its health impact on mothers and fathers. Review studies have shown that the strain caused by work-family conflict leads to poorer health (16, 17). This can be reflected both in mental and in physical health (14, 16–18). More recently, stable associations between both directions of work-family conflict and a large number of health parameters have been revealed. Among others, results were presented on self-rated health, psychological strains, somatic and depressive symptoms (19). Other studies show an association to physical health, health-related behavior, and sleeping patterns (20). However, most of these studies are publications based on US data. Although it is known that the interrelation of work-family conflict and health varies with different political and societal contexts, the transferability of the results described above to the European area has not been studied to date.

In addition to the aforementioned state of research, the impact of work-family conflict on health may differ depending on the subgroups and directions of conflicts studied. On average, mothers spend more time doing unpaid work than fathers, irrespective of their employment status and fathers usually spend more hours per week in paid employment (21). Studies show that mothers and fathers perceive work-family conflict differently (21, 22). The available results on differences between effects of work-family conflict on the health working mothers and fathers are, however, rather controversial and do not provide unequivocal information (3, 21, 24). Other social determinants, such as profession, education and income, have only rarely been taken into account by the available publications on work-family conflict and health, although this has already been called for from various quarters (4, 16).

Furthermore, there is no clarity regarding the associations between the two directions of work-family conflict, work-to-family and family-to-work conflict, and health outcomes. In the case of mental health, on the one hand, there is evidence suggesting that both conflict directions are associated with the outcome (17, 23). The picture is similar in self-rated health, although, on the other hand, a small percentage of the studies shows no association with the two conflict directions (17). Here, too, there is a lack of review studies examining the conflict directions systematically (16, 25).

### Objectives

The review aims at the identification and analysis of knowledge gaps regarding health-related consequences of work-family conflicts in Europe. As most of the existing studies in the field are publications based on US data, the goal is to show the scope of the European state of research since 2000. Thus, the authors aim at identifying the available and relevant European studies on work-family conflict and health and will map, report and discuss the results of these studies. In doing so, they will be able to identify relevant fields and connecting points for future public health research on the presented topic.

### Research Question

The review examines the following research questions:

1. What data sets, samples, study designs, and survey instruments are used to investigate associations between work-family conflict and health?
2. What associations are revealed between work-family conflict and health in Europe?
3. Are systematic differences relating to the directions of conflict, gender, or social determinants recognizable in the association of work-family conflict and health?

## Methods

### Study Design

A scoping review approach is applied. In line with the objectives outlined above, the application of a scoping review approach is indicated to determine the scope of a body of literature on a given topic. The rationale for choosing a scoping review over a systematic review is to map the available and relevant evidence on this respective topic instead of e.g., assessing the effectiveness of a certain treatment or practice (26–28). The research was carried out by closely following the Arksey and O'Malley methodological framework for conducting a scoping study (26, 28).

### Search Strategy and Data Sources

**Figure 1:**
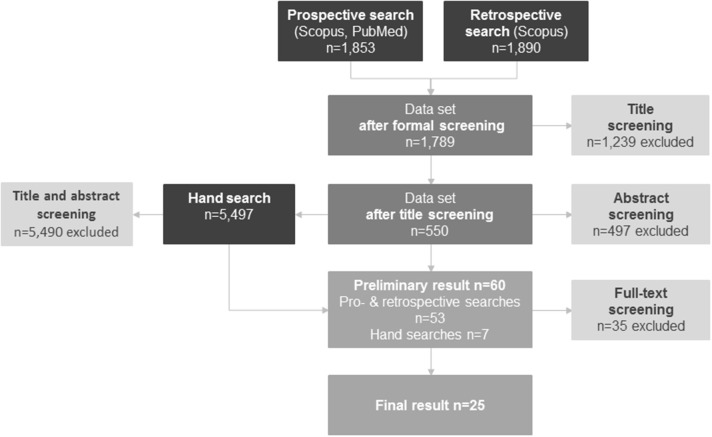
PRISMA diagram of search strategies.

Common scoping review searches include multiple sources of data and a combination of search strategies (26, 28). To account for the complexity of the topic and for the several scientific disciplines involved, this review combined a prospective search based on landmark publications with supplementary keyword and hand searches in databases (see Figure 1) to find relevant articles that were potentially missed in the database searches (29).

#### Main Search Strategy and Data Sources

The main search was based on forward citation tracking on the basis of four landmark publications. Landmark publications are research works which—in the researcher's view and according to the assessment of experts—are key publications on a chosen topic. They are often themselves reviews (30). When selecting the four landmark publications for this review (4, 16, 17, 19), care was taken to ensure that they had a clear reference to the topic of health-related outcomes of work-family conflict. The publications were to stem from different scientific disciplines that are relevant to the thematic area to be examined (25). In the present paper this applied, among others, to the scientific fields of sociology, psychology, and public health. A prospective search in the PubMed and Scopus databases incorporated all the literature from the years 2000 to 2017 that cited the landmark publications. In addition to a focus on medical topics, PubMed also yields a large selection of psychological topics; Scopus was used due to its wide range of social science literature. A total of *n* = 1,853 search results were included. The procedure was supplemented by a retrospective search in the Scopus database in the areas of title, keywords and abstract for the years 2000 to 2017 using an English-language search string (see Appendix A). This search generated *n* = 1,890 search results.

#### Additional Searches and Data Sources

Hand searches were carried out in selected journals, the publication lists of research networks, and the Google and Google Scholar search engines to supplement the results of the main search (29). These resulted in a total of *n* = 5,497 hits. All European journals that contributed at least three publications to the data set of the main search after title-screening were included (European Journal of Public Health, Scandinavian Journal of Psychology) and entries published between January 2000 and December 2017 were searched (*n* = 4,698 contributions). After screening of titles and abstracts *n* = 2 papers were included. Furthermore, the publication lists of relevant research networks (European Institute of Women's Health, I.Family Study, Kanter Award for Excellence in Work-Family Research, Work, Family & Health Network, Work and Family Researchers Network) were screened applying the inclusion and exclusion criteria. The networks were selected on the basis of mentions within the results identified by the main search. A total of *n* = 599 entries were studied. After title and abstract screening, *n* = 2 contributions were included in the publication lists. In addition, the first *n* = 200 hits were analyzed in Google and Google Scholar using a simplified search string. After title and abstract screening, *n* = 3 relevant contributions were included.

### Inclusion and Exclusion Criteria, Study Sections, and Data Extraction

The following inclusion and exclusion criteria were defined for the selection of relevant publications and applied in the screening of titles and abstracts as well as the sections “materials and methods” and “results” in the full-text screening (Table 1).

**Table 1:** Inclusion and exclusion criteria.

	**Inclusion**	**Exclusion**
Language	German, English	Other languages
Year of publication	2000 to 2017	1999 and earlier
Region	WHO-EURO	Other regions
Study type	- Research articles showing empirical results	- Overview articles - Commentaries - Corrections - Evaluations
Study focus	- Impact of work-family conflict - Health outcomes	-Causes of work-family conflict -Other outcomes
Age of persons studied	Adults aged between 18 and 49	Children and young people under 18, adults aged 50 and older
Work-family interface	- Work-family conflict - Work family interference - Work-family spillover	- Work-family enrichment - Work-family balance - Crossover - Work-life and work-home concepts
Health outcomes	- Self-rated health - Mental health - Physical health - Health-related behavior - Sleep - Health services utilization	- Stress - Burnout - Well-being - Exhaustion - Satisfaction with life - Sick days - Occupational accidents

To set a focus on work-family conflict, the minimum requirement for “family” was the consideration of the number of children in the household as a control variable. Moreover, age was limited to 18 to 49 in order to further narrow down a focus on parents, as opposed to relatives providing nursing care. Concepts of positive associations between working and family roles (“enrichment” and “balance”) were also excluded in order to maintain the focus on health risks. Studies with outcomes such as burn-out, stress, and exhaustion were excluded from the sample. This is justified since the nature of these outcomes is often measured as job-related (instead of health-related); also, in some cases, the conceptualizations of these outcomes yield at similar characteristics as the concept of work-family conflict itself and can thus not be clearly distinguished. Furthermore, due to the prospective nature of the main search, the lower limit of the studied time span was set to the year of 2000 as the least recent landmark publication was published in this year. The data extraction was carried out systematically by applying a data-charting form for each study.

### Data Analysis

The evaluation is carried out as a narrative synthesis of the results from the *n* = 25 publications. The key results of the individual studies are reported in groups along the health outcomes reported in the publications. In addition to methodological aspects of the individual publications, the focus here is on presenting the results on the association between work-family conflict and health. The direction of work-family conflict (work-to-family conflict or family-to-work conflict) is taken into account, as are differentiated results by gender and other social determinants.

## Results

### Sample and Study Characteristics

**Figure 2:**
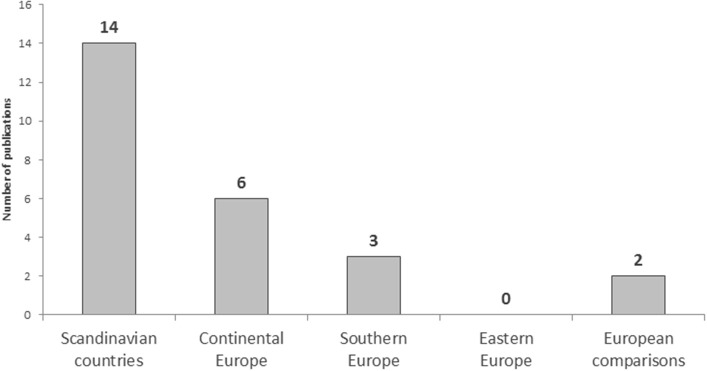
Number of publications by region, 2000–2017, *n* = 25.

The *n* = 25 articles were published between 2004 and 2017. The results are most common from Scandinavia (Finland, Norway, and Sweden) and continental Europe (Austria, Belgium, Germany, the Netherlands, and Switzerland, see Figure 2). By contrast, data from the south of Europe (Italy, Portugal, and Spain) and inter-European comparisons are relatively infrequent. No publications from eastern European countries are included in the sample.

A look at the health outcomes reveals that more than 40% (*n* = 12) of the publications examine the association of work-family conflict and mental health. A further seven studies focus on subjective health, which was usually recorded as self-rated general health. Twenty percentage of the studies (*n* = 5) examine symptoms of physical health, and three publications consider the outcome sleep. Only isolated results are available (total *n* = 2) on health-related behavior and health services utilization.

Fifteen different measurement scales are used to survey work-family conflict. In some cases, these instruments have been even further adapted to the respective survey context. The number of items on the individual scales ranges from a single-item scale to the 36-item long instrument developed by Frone et al. (31).

With regard to the conflict directions described, nine studies make no distinction between work-to-family conflict and family-to-work conflict. Four further studies distinguish between the directions, but refer only to work-to-family conflict while 12 publications refer to both conflict directions.

Eight of the 25 publications are based on population-representative data. The remaining 17 studies examine the association between work-family conflict and health by looking at employees of individual companies or specific occupational groups such as police officers, public servants and various groups of medical staff.

### Outcomes of Health in Working Mothers and Fathers

#### Mental Health

The most frequently available results (twelve publications) on the health effects of work-family conflict deal with mental health (Table 2, lines a-l). The greatest methodological diversity can also be observed here: five analyses were implemented on the basis of longitudinal data and four studies worked with population-representative data sets. The studies come from continental and southern Europe, as well as from Scandinavia, and also include European comparisons.

**Table 2:** Selected publications for review.

	**Author**	**Year**	**Title**	**Country**	**Population**	**Outcome**	**Work-family conflict**	**Data type**
a	du Prel JB, Peter R	2015	Work-family conflict as a mediator in the association between work stress and depressive symptoms: cross-sectional evidence from the German lidA-cohort study	Germany	National sample representative of employees except self-employed, freelancer, and civil servants	Mental health	WtFC	C
b	Haggag AK, Geser W, Ostermann H, Schusterschitz C	2012	Relation of work family conflict and role quality on depressive symptoms in mothers	Austria	National sample representative of mothers	Mental health	WtFC, FtWC	C
c	Jensen MT, Rundmo T	2016	Associations between work family conflict, emotional exhaustion, musculoskeletal pain, and gastrointestinal problems in a sample of business travelers	Norway	Employees from two units of an oil and gas company	Physical health	n.i.	C
d	Jensen MT, Knudsen K	2017	A two-wave cross-lagged study of business travel, work–family conflict, emotional exhaustion, and psychological health complaints	Norway	Employees from two units of an oil and gas company	Mental health	n.i.	L
e	Kinnunen U, Feldt T, Geurts S, Pulkkinen L	2006	Types of work-family interface: Well-being correlates of negative and positive spillover between work and family	Finland	Participants of the Jyväskylä student cohort	Mental health	WtFC, FtWC	C
f	Mikkelsen A, Burke RJ	2004	Work-family concerns of Norwegian police officers: Antecedents and consequences	Norway	Sample of police officers	Self-rated health, mental health	n.i.	C
g	Moreno-Jiménez B, Mayo M, Sanz-Vergel AI, Geurts S, Rodríguez-Muñoz A, Garrosa E	2009	Effects of work-family conflict on employees' well-being: The moderating role of recovery strategies	Spain	Sample of emergency professionals	Mental health	WtFC, FtWC	C
h	Neto M, Carvalho VS, Chambel MJ, Manuel S, Miguel JP, De Fatima Reis M	2016	Work-family conflict and employee well-being over time. The loss spiral effect	Portugal	Employees of a company	Mental health	n.i.	L
i	Rantanen J, Pulkkinen L, Kinnunen U	2005	The big five personality dimensions, work-family conflict, and psychological distress: A longitudinal view	Finland	Participants of the Jyväskylä student cohort	Mental health	WtFC, FtWC	L
j	Rantanen J, Kinnunen U, Feldt T, Pulkkinen L	2008	Work-family conflict and psychological well-being: Stability and cross-lagged relations within one- and six- year follow-ups	Finland	National sample representative of employed citizens in a relationship and/or with children	Mental health	WtFC, FtWC	C
k	Symoens S, Bracke P	2015	Work-family conflict and mental health in newlywed and recently cohabiting couples: A couple perspective	Belgium	Regional sample representative of newlywed and recently cohabiting heterosexual couples	Mental health	WtFC, FtWC	C
l	Kinnunen U, Geurts S, Mauno S	2004	Work-to-family conflict and its relationship with satisfaction and well-being: A one year longitudinal study on gender differences	Finland	National sample representative of employed citizens in a relationship and/or with children	Mental health, physical health	WtFC	L
m	Cullati S	2014	The influence of work-family conflict trajectories on self-rated health trajectories in Switzerland: A life course approach	Switzerland	National sample representative of employed citizens	Self-rated health	WtFC	L
n	Christiaens W, Bracke P	2014	Work-family conflict, health services and medication use among dual-income couples in Europe	Europe (Austria, Belgium, Switzerland, the Czech Republic, Germany, Denmark, Estonia, Spain, Finland, France, the UK, Greece, Hungary, Ireland, Iceland, Italy, Luxembourg, The Netherlands, Norway, Poland, Portugal, Sweden, Slovenia, the Slovak Republic)	National samples representative of citizens living in private households	Self-rated health, health services utilization	WtFC, FtWC	C
o	Leineweber C, Baltzer M, Magnusson Hanson LL, Westerlund H	2013	Work-family conflict and health in Swedish working women and men: a 2-year prospective analysis (the SLOSH study)	Sweden	National samples representative of employees living together with a (heterosexual) partner	Self-rated health, health-related behavior	n.i.	L
p	Tunlid S	2014	Work-family conflict in Sweden and Germany: A study on the association with self-rated health and the role of gender attitudes and family policy	Europe (Germany, Sweden)	National samples representative of citizens living in private households	Self-rated health	WtFC, FtWC	C
q	Väänänen A, Kevin MV, Ala-Mursula L, Pentti J, Kivimäki M, Vahtera J	2005	The double burden of and negative spillover between paid and domestic work: Associations with health among men and women	Finland	Sample of full-time employees from 10 towns	Self-rated health	WtFC, FtWC	C
r	Winter T, Roos E, Rahkonen O, Martikainen P, Lahelma E	2006	Work-family conflicts and self-rated health among middle-aged municipal employees in Finland	Finland	Employees of the City of Helsinki	Self-rated health	WtFC, FtWC	C
s	Höge T	2009	When work strain transcends psychological boundaries: An inquiry into the relationship between time pressure, irritation, work-family conflict and psychosomatic complaints	Germany	Female home care nurses with at least one child	Physical health	n.i.	C
t	Jensen MT	2016	A two wave cross-lagged study of work-role conflict, work-family conflict and emotional exhaustion	Norway	Employees from two units of an oil and gas company	Mental health	n.i.	L
u	Mauno S, Kinnunen U, Ruokolainen M	2006	Exploring work- and organization-based resources as moderators between work-family conflict, well-being, and job attitudes	Finland	Employees from three companies (healthcare district, ICT company, carton board mill)	Physical health	WtFC	C
v	van Steenbergen EF, Ellemers N	2009	Is managing the work-family interface worthwhile? Benefits for employee health and performance	The Netherlands	Employees from a multinational financial services organization	Physical health	WtFC, FtWC	C
w	Vedaa O, Krossbakken E, Grimsrud ID, Bjorvatn B, Sivertsen B, Magerøy N, Einarsen S, Pallesen S	2016	Prospective study of predictors and consequences of insomnia: Personality, lifestyle, mental health, and work-related stressors	Norway	Employees from three organizations	Sleep	WtFC, FtWC	L
x	Mäkelä L, Bergbom B, Tanskanen J, Kinnunen U	2014	The relationship between international business travel and sleep problems via work-family conflict	Finland	Sample of registered members of the Norwegian Nurses Organization	Sleep	n.i.	L
y	Camerino D, Sandri M, Sartori S, Conway PM, Campanini P, Costa G	2010	Shiftwork, work-family conflict among Italian nurses, and prevention efficacy	Italy	Regional sample representative of of healthcare nurses	Sleep	n.i.	C

*WtFC, work-to-family conflict; FtWC, family-to-work conflict; n.i., not indicated; C, cross-sectional data; L, longitudinal data*.

The results show that work-family conflict and poorer mental health are related (e), although the effects are sometimes weak (f). This was reported for both conflict directions (work-to-family conflict and family-to-work conflict) (g), as well as for the specific outcome depression (a, b, k). In the case of emotional exhaustion (c) and psychological well-being (h) it was shown that poorer mental health also determines the incidence of work-family conflict (reverse causality). However, the picture remains heterogeneous as in another study work-family conflict predicts emotional exhaustion, but not other psychological symptoms (d). Similarly, a further longitudinal analysis found no associations after 1 year and after 6 years (i).

Moreover, one study shows that a causal association with mental health only exists in mothers. For fathers, however, the relationship was very strong in cross-section (e). Mothers are also more strongly affected in both directions while in fathers family-to-work conflict is more strongly associated with depression than work-to-family conflict (k).

The extent to which the association between work-family conflict and mental health is moderated by other social determinants was not investigated in the available studies.

#### Self-Rated Health

Seven studies in the examined sample were published on self-rated health (f, m–r). Four were conducted on the basis of population-representative surveys and two studies applied longitudinal data. The examined data originates from Scandinavia and continental Europe.

Four studies found associations between the two directions of work-family conflict and self-rated health (p–r). One study published results on the reverse causal direction, so that poorer self-rated health could predict higher work-family conflict (o). However, two other studies showed no direct association (f, m).

A differentiated view by gender draws an uneven picture. In a publication from Germany, for example, both conflict directions are most closely related to self-rated health in fathers (p). In other publications, however, family-to-work conflict is only for mothers associated with poorer self-rated health (n, q). As regards family-to-work conflict, another study concludes that mothers report stronger effects on self-rated health than fathers (o).

Results on other social determinants reveal that better material resources (r) and a higher level of education (m) reduce the negative effect of high work-family conflict on self-rated health.

#### Physical Health

A total of five publications are available in the field of physical health (l, s–v). All but one study report results based on specific populations. One longitudinal analysis was carried out (l) that also allows causal conclusions to be drawn. The publications come from Scandinavia and continental Europe.

The results show that work-family conflict and psychosomatic symptoms are associated (s), and that strain-related conflicts have a greater effect than time-related conflicts (u). In addition, work-to-family conflict is associated with higher cholesterol, obesity and low physical fitness, but family-to-work conflict is not (v). However, other studies do not show any associations between work-family conflict and physical health, or else the association is fully explained by emotional exhaustion (t).

No differences are shown between mothers and fathers (l). None of the studies considers other social determinants of health than gender.

#### Other Outcomes: Sleep, Health-Related Behavior, Health Services Utilization

There are five studies on other health outcomes; they consider sleep, health-related behavior and the health services utilization.

All three studies available on sleep (w–y) examine specific populations (nurses and business travelers) in Scandinavia and southern Europe. They come to the conclusion that work-family conflict and poor sleep are related. One publication based on longitudinal data shows that sleep and work-family conflict are related in both causal directions (x). The articles either do not distinguish between the directions of conflict or only investigate work-to-family conflict. Furthermore, no specific results are available for differences by gender or other social determinants.

The study on health-related behavior (o) examines the longitudinal association between work-family conflict and alcohol consumption among working mothers and fathers in Sweden, with fathers reporting increased alcohol consumption as a consequence of work-family conflict.

One study with data from the European Social Survey is available on the utilization of health services (n). It shows that work-to-family conflict leads to an increase in health services utilization and a higher medication intake in mothers, but that family-to-work conflict does not.

## Discussion

### Summary of Main Findings

The results of research question 1 show that to date no studies are available for countries in Eastern Europe. Inner-European comparisons have rarely been carried out as the association between work-family conflict and health is mainly studied in individual countries. In addition, less than half of the examined studies used longitudinal study designs or carried out analyses based on population-representative samples. Also, a large number of different measuring instruments are used to survey work-family conflict and the concepts are not always clearly operationalized and theoretically defined.

The results on research question 2 confirm the associations already found in US-based review studies between work-family conflict and mental, self-rated, and physical health. However, sleep, health-related behavior and the utilization of health services were examined in only a few publications in Europe. The overall picture of the studies suggests the interpretation that work-family conflict and mental, self-rated and physical health are interrelated.

With regard to research question 3, an association was reported for mental and self-rated health and both work-to-family conflict and family-to-work conflict; in the case of physical health, only associations with work-to-family conflict could be shown. There is also a heterogeneous picture when it comes to differences between working mothers and fathers in the association between work-family conflict and health. In the case of mental and self-rated health, some studies have shown that mothers are affected by both conflict directions, but fathers only by work-to-family conflict. Other publications can show an association between both conflict directions and self-rated health, also for fathers. On the other hand, no differences can be seen between mothers and fathers in the field of physical health. Further differentiation characteristics are only considered for self-rated health and show that a better material situation and a higher level of education weaken the link between work-family conflict and poorer health.

### Classification of the Results

The results predominantly show an association between work-family conflict and health. This is in line with the findings of review studies, in which work-family conflict correlates with mental, physical and self-rated health as well as health-related behavior (4, 16, 17, 19, 23). However, since most of the review studies are drawn on US-based data and publications, the present study can provide the first comprehensive overview of the links between work-family conflict and health in the European region. This seems particularly relevant as social security systems differ between the US and most of the European countries, particularly in terms of paid parental leave and the availability of state-provided institutional childcare (32).

Yet an analysis of this review's results leaves a number of questions unanswered. It remains unclear, for which conflict direction (work-to-family conflict or family-to-work conflict) associations between work-family conflict and health exist. Furthermore, the extent to which working mothers and fathers are equally burdened by the health effects of work-family conflict remains an open question and there are also still only sporadic results that examine differences in the health effects of work-family conflict as a function of social determinants. Not only in this article, but also in the international research context of work-family conflict and health, it has not been possible to answer these questions unequivocally and consistently across several health outcomes (17).

However, the results obtained in this review on the methodological approaches of the considered studies may provide possible explanations for this. On the one hand, the available articles contain considerable variations in the measurement of work-family conflict and the samples investigated. This heterogeneity almost precludes any comparison between the different studies and can thus explain some of the controversial results (16). In addition, an examination of the European state of research on the subject reveals the perspective of—perhaps considerable—variations between countries primarily caused by the political conditions for reconciling work and family roles (23). Moreover, a mere differentiation according to gender may not be enough to identify groups that are under particular strain. Rather, further subgroups need to be formed using combinations of characteristics like gender and social determinants of health. These three perspectives are discussed below.

The many different survey instruments used for work-family conflict can lead to different results on associations between work-family conflict and health (15, 16). Moreover, many of the scales used have not been validated for the European region, making it difficult to compare results between different European countries and with non-European research findings (15). The focus on cross-sectional analyses suggested by the available data means that causal association paths have not yet been considered in a sufficiently differentiated way. Since only less than a third of the publications use a population-representative database, results from studies on specific study populations have usually not been reproduced with population-representative data (16, 17, 20, 33). As the majority of studies examine these specific populations such as individual occupational groups, employees of a specific organization, or participants in a highly localized cohort study, the comparability of the results can be hampered. Particularly the specific political and regulatory conditions in the respective employment context can have a major impact on the incidence and health-related outcomes of work-family conflict (34).

The differences in study design, sampling procedure and measurement concepts make it difficult to clearly identify the causes of controversial results. This also applies to an analysis of the political conditions regarding the reconciliation of work and family lives. A review study on this subject shows on the one hand that differences in the incidence of work-family conflict among working mothers and fathers vary between countries. On the other hand, no clear factors could be found here to explain these differences. This, too, is attributed, among other things, to the heterogeneous survey methods (32).

However, with the help of country comparisons, individual studies also specifically examine the role of political conditions in the association between work-family conflict and health. It has been shown that effects from political and cultural contexts moderate differences in the association between work-family conflict and health (21, 35). However, the results are not unequivocal in all studies. On the one hand, mothers in countries with more conservative family policies report poorer self-rated health compared to fathers when there is a work-family conflict. On the other hand, this difference is less pronounced in countries where dual-earner strategies are politically supported, or where there is little government intervention in the organization of work and family lives (35). These results are partly supported by Artazcoz et al. who could not find any health effects of work-family conflict in Nordic countries that politically support dual-earner models and a high level of equality between working mothers and fathers (36). Similarly, Hagqvist et al. (21) report that work-family conflict occurs less frequently in countries with a high level of politically and socially supported equality among working mothers and fathers. However, the association between work-family conflict and poorer health was stronger in these countries than in countries that support more traditional family models (21). So although here, too, no clear patterns can be discerned, the findings nevertheless indicate that, policy measures regarding the reconciliation of work and family lives and gender norms can be related to the population's health. However, the differentiated mechanisms of this link will have to be examined more closely in future research.

The inclusion of political conditions is an important way of looking at the respective contexts in which work-family conflict occurs and causes health problems. However, in order to more thoroughly clarify the heterogeneous results on the health effects of work-family conflict, further variables that go beyond context should be included in the analyses (16, 37). There are calls from several sides, for example, for greater consideration to be given to characteristics of the individual socioeconomic position and in particular to focus on employees in lower-status educational and occupational groups (4, 16, 33). This is particularly relevant since studies focus mainly on middle-class employees or more highly educated and skilled occupational groups up to now. Preparatory work on this has shown that work-family conflict can help explain social differences in health. However, the results of this review on Europe show that up to now only few publications relate associations between work-family conflict and health to differences in education, financial position, and social status in general. In order to meaningfully integrate these social determinants into models, greater consideration should be given to a so-called intersectional perspective (37). Intersectional means that social determinants, such as gender and education, should not only be considered as individual control variables, but that interactions between the determinants and resulting subgroups should be included in the analyses. The application of intersectional perspectives can be expected to enable subgroups with specific health burdens caused by work-family conflict to be identified more clearly than it would be possible with one-dimensional consideration of individual characteristics. The intersectional perspective regards gender differences as dynamic constructs that vary, for example, with the political and social context as well as with a person's individual socioeconomic position (37).

### Limitations

When interpreting the results presented, the following methodological limitations must be taken into account. The researchers themselves identified relevant publications on the basis of the defined inclusion and exclusion criteria. This is also practiced elsewhere in the context of scoping review methods (38). Also, as scoping reviews rather aim at providing an overview of the evidence instead of answers to questions from a narrow range of publications, a systematic assessment of the quality of the included studies as well as a risk of bias assessment were not carried out, as suggested by Munn et al. (27).

Furthermore, only publications in German and English were included in the database. Although the majority of studies in the social and natural sciences are currently published in English, this could mean that some studies from individual European countries have not been included in the database.

### Conclusion and Implications for Future Research

Besides the aforementioned limitations, the present review provides the first comprehensive overview of the state of research on work-family conflict and health in Europe.

However, the mapping of studies on work-family conflicts and health in Europe revealed methodological and content-related implications for future research. On the one hand, more longitudinal analyses are necessary to validate the partially controversial results published to date in studies on the causal association between work-family conflict and health (16, 17).

Furthermore, it would be useful if standardized instruments for the measurement of work-family conflict were used more extensively in population-based studies. This would facilitate the comparability and transferability of results and make more valid statements possible (16, 17, 32).

In terms of content, on the other hand, Frone et al. (23) already called for the consideration and incorporation of the cultural, political and societal contexts in which work-family conflict emerges and affects health (23). Although initial research results in this field are already available, however, they suggest that the focus should be on the roles and interplay of political measures and gender norms in the population (37).

There is also a lack of results on the association between work-family conflict and health in eastern European countries. These would be helpful in understanding changes in gender norms since the collapse of the Soviet Union and any effects these changes might have had (39).

In order to gain a better understanding of the health-related burdens caused by work-family conflict in different societal groups, it remains necessary to implement analyses on a large number of study populations: for example, the analyses should focus on financially disadvantaged groups, single mothers and fathers in particular, as well as people who nurse relatives (4, 16). It is important in this context to also use interactions between social determinants in the sense of intersectional research approaches (37, 40). By observing the subgroups that emerge from combining factors, the quality and thus the informative value of the research can be increased, especially with regard to the heterogeneity of effects and causal processes (40).

## Data Availability

The data set of articles supporting the conclusions of this manuscript will be made available by the authors, without undue reservation, to any qualified researcher.

## Author Contributions

Conceptualization, methodology, and writing original draft: L-SB. Supervision: TL. Writing review and editing: L-SB, PR, and TL.

### Conflict of Interest Statement

The authors declare that the research was conducted in the absence of any commercial or financial relationships that could be construed as a potential conflict of interest.

## Appendix A

TITLE-ABS-KEY (((“work family balance”) OR (“work family conflict”) OR (“work family interference”) OR (“work family spill over”) OR (“work family spillover”) OR (“work family spill-over”) OR (“work home balance”) OR (“work home conflict”) OR (“work home interference”) OR (“work home spill over”) OR (“work home spillover”) OR (“work home spill-over”) OR (“work life balance”) OR (“work life conflict”) OR (“work life interference”) OR (“work life spill over”) OR (“work life spillover”) OR (“work life spill-over”)) AND (depression OR disease OR health OR stress)) AND (LIMIT-TO (PUBYEAR, 2018) OR LIMIT-TO (PUBYEAR, 2017) OR LIMIT-TO (PUBYEAR, 2016) OR LIMIT-TO (PUBYEAR, 2015) OR LIMIT-TO (PUBYEAR, 2014) OR LIMIT-TO (PUBYEAR, 2013) OR LIMIT-TO (PUBYEAR, 2012) OR LIMIT-TO (PUBYEAR, 2011) OR LIMIT-TO (PUBYEAR, 2010) OR LIMIT-TO (PUBYEAR, 2009) OR LIMIT-TO (PUBYEAR, 2008) OR LIMIT-TO (PUBYEAR, 2007) OR LIMIT-TO (PUBYEAR, 2006) OR LIMIT-TO (PUBYEAR, 2005) OR LIMIT-TO (PUBYEAR, 2004) OR LIMIT-TO (PUBYEAR, 2003) OR LIMIT-TO (PUBYEAR, 2002) OR LIMIT-TO (PUBYEAR, 2001) OR LIMIT-TO (PUBYEAR, 2000)).
